# Linguistic Skill and Stimulus-Driven Attention: A Case for Linguistic Relativity

**DOI:** 10.3389/fpsyg.2022.875744

**Published:** 2022-05-20

**Authors:** Ulrich Ansorge, Diane Baier, Soonja Choi

**Affiliations:** ^1^Faculty of Psychology, University of Vienna, Vienna, Austria; ^2^Cognitive Science Hub, University of Vienna, Vienna, Austria; ^3^Research Platform Mediatised Lifeworlds, University of Vienna, Vienna, Austria; ^4^Acoustics Research Institute, Austrian Academy of Sciences, Vienna, Austria; ^5^Department of Linguistics and Asian/Middle Eastern Languages, San Diego State University, San Diego, CA, United States; ^6^Faculty of Philological and Cultural Studies, University of Vienna, Vienna, Austria

**Keywords:** language, attention, linguistic relativity, visual saccade, automatic processing

## Abstract

How does the language we speak affect our perception? Here, we argue for linguistic relativity and present an explanation through “language-induced automatized stimulus-driven attention” (LASA): Our respective mother tongue *automatically* influences our attention and, hence, perception, and in this sense determines what we see. As LASA is highly practiced throughout life, it is difficult to suppress, and even shows in language-independent non-linguistic tasks. We argue that attention is involved in language-dependent processing and point out that automatic or stimulus-driven forms of attention, albeit initially learned as serving a linguistic skill, account for linguistic relativity as they are automatized and generalize to non-linguistic tasks. In support of this possibility, we review evidence for such automatized stimulus-driven attention in language-independent non-linguistic tasks. We conclude that linguistic relativity is possible and in fact a reality, although it might not be as powerful as assumed by some of its strongest proponents.

## Introduction

According to the concept of linguistic relativity, the language a human speaks shapes her/his perception (for recent reviews of the evidence, see Athanasopoulos and Casaponsa, 2020; Lupyan et al., 2020). To put it in the words of Sapir, we humans “see and hear and otherwise experience very largely as we do because the language habits of our community predispose certain choices of interpretation” (Sapir, 1941/1964, p. 69; see also Whorf, 1956). We propose that stimulus-driven attention accounts for some of these profound influences of language on perception, specifically with our currently proposed model, which we call “language-induced automatized stimulus-driven attention” (LASA).

Within the framework supporting “linguistic relativity,” researchers have proposed different claims (Slobin, 1996; Majid et al., 2004) in the degree to which language directly influences our perception. As will be shown, our proposed model is closer to some recent views supporting linguistic relativity, but contrasts with others. Importantly, our model goes further than any current view by incorporating “automatization” – from the stimulus-driven level – in the mechanism of language shaping perception and explaining in detail how it works.

Our view aligns with a strong version of linguistic relativity, which drew on the concept of attention to argue for linguistic relativity, such as Majid et al. (2004) or Thierry et al. (2009). Majid et al. (2004) for example, noted that languages show different preferred spatial frames of references for spatial object relations, such as absolute positions (e.g., “to the North” in Guugu Yimithirr, even for the locations of “table-top” objects, such as the position of a spoon relative to plate) versus relative positions (e.g., “to the left” in Dutch, for the same relations). Majid et al. (2004) observed that speakers of such different languages showed the consistent language-specific preferences for particular frames of references when solving non-linguistic object-arrangement tasks. Moreover, these authors speculated that attention – the human ability to select some object, feature, location, or sense, while at the same time ignoring others – could be responsible for these generalizations of “linguistic habits” to non-linguistic tasks. According to this idea, for example, speakers of Tzeltal, another language with a preference for absolute frames of references, would in general pay attention to update locations of objects in their surroundings in terms of [or “following”] the absolute frame of references.

However, our argument is in contrast to widely held but weak version of linguistic relativity, namely that the influences of linguistic specifics on attention only play out during verbal processing itself – that is, while speaking, reading, writing, or verbally comprehending (e.g., Slobin, 1996, 2000, 2003). Slobin (1996, p. 89) admitted that during communication, attention can play a decisive role for what is represented: “In brief, each native language has trained its speakers to pay different kinds of attention to events and experiences when talking about them.” However, Slobin restricted these influences to linguistic processing (e.g., Slobin, 2003, p. 159): “I wish to argue that serious study of *language in use* points to pervasive effects of language on selective attention and memory for particular event characteristics. As I’ve argued in greater detail elsewhere (Slobin, 1996, 2000), whatever effects language may have when people are not speaking or listening, the mental activity that goes *on while formulating and interpreting utterances* is not trivial or obvious, and it deserves attention” [italics added by us]. In conclusion, Slobin (1996; 2000; 2003), clearly argues that *use of language* is involved in mental activity and it is such “covert” language use that influences our non-linguistic cognition during communication.

The question of whether *attention that derives from language-specific grammar* generalizes to non-linguistic tasks in a pervasive/direct way and, therefore, allows for linguistic relativity, is contested until today, as hitherto the evidence is not that clear. For example, critics could argue that observers in the studies on spatial frames of references reviewed by Majid et al. (2004) were free to use linguistic means to solve their spatial tasks. At the very least, language might be helpful when an observer has to decide where to put one object relative to another one as was required in the spatial puzzles reviewed in Majid et al. (2004). Likewise, proof of linguistic influences on attention in linguistic tasks as provided by Slobin (1996; 2000; 2003), does not rule out that these linguistic influences on attention would not also be present in non-linguistic tasks. The current perspective, thus, seeks to explain how language-dependent attentional selection exerts its effects in non-linguistic tasks.

## Language as a Skill

To start with, languages are symbolic systems for communicative purposes (cf. Clark and Clark, 1977). Its symbols are different from, but referring to such diverse things as ideas, objects, and events. Take the example of the word *bread*. It refers to a piece of bread as an object but is clearly different from the particular shape or material of the object. Language is also conventional. Its meaning, use, and structure are governed by the rules shared by the many people who speak it. For instance, only if people consent upon the meaning of the word *bread*, can this word be used to successfully refer to this object and can the word be correctly understood in a conversation. Language is also structured on many different levels from phonemes and letters via words, sentences, and texts, to turn-taking and discourses.

Critically, language execution – whether in the form of speaking, reading, or auditory comprehension – is based on acquired skills (e.g., Stark, 1980; Afflerbach et al., 2008; DeKeyser, 2020). Starting from an early age, humans practice their mother tongue for years. Skills consist of a number of different actions (e.g., with different effectors) and covert processes that are jointly executed in an integrated fashion, for example, with particular sequences and with switches back and forth between gross and fine motor actions or between overt motor actions and covert processing (e.g., Newell and Rosenbloom, 1981; Anderson et al., 2004). Think of reading as an example. The eyes move overtly across the text by jumping movements, so-called saccades, and intermittent fixations – that is, times during which the eyes are relatively still. Without much effortful monitoring, these overt eye movement actions smoothly take turns with covert processes of lexical and phonological processing of inputs, mostly occurring during fixations (cf. Reichle et al., 2003; Engbert et al., 2005). There is evidence that, during fixations, covert processing steps such as lexical processing occur as indicated by both behavioral as well as electrophysiological evidence (e.g., Sereno and Rayner, 2003; Dimigen et al., 2011). For example, fixations tend to land on words carrying the bulk of the information rather than on mere functional words such as articles. Fixations are also typically longer for less than for more frequent words and for ambiguous than for unambiguous words (Duffy et al., 1988; Rayner et al., 1996). All of these findings indicate that, during fixations, information from the words is covertly processed, either about the currently fixated word or about the potential candidate words for the next fixation (or landing point).

Reading, speaking, or verbal comprehension are all skills. As a consequence of their regular practice, skills are represented in human long-term memory (e.g., Johnson, 2013). If sufficiently well-trained, they are executed automatically with high efficiency, meaning that they do not need much top-down control, active monitoring, or willing deliberation to start with (e.g., d‘Ydewalle et al., 1991; Feng et al., 2013). Instead, it is typical for skills to proceed automatically, at least in part, meaning that control is delegated to the fitting stimuli, and that the stimuli themselves can trigger the skill (cf. Reason, 1990). As an example, think of hearing someone calling your own name. Even if you are entirely occupied with doing something else, hearing your own name is often distracting (cf. Perrin et al., 1999; Röer et al., 2013). It can capture your attention and trigger verbal comprehension, though you might have been engaged in an entirely non-linguistic task, such as painting or running.

## Language Skills and Visual Attention

Visual attention is critical part and parcel of most skills, including language skills (e.g., Johnson, 2013). In this context, *attention* denotes the selection of information for its use in action control (*sic*!) and covert processing (e.g., encoding information into memory or retrieving information from memory; *sic*!). *Visual attention* denotes the corresponding selections of information from the visual environment. That visual attention is involved in language skills is obvious in the case of reading. For example, for the successful fixation of the next word during reading, humans need to select the position of this word for the programming of a saccade with a fitting direction and amplitude (cf. Reichle et al., 2003; Engbert et al., 2005). In addition, in line with a high degree of automaticity, words seemingly attract visual attention automatically. For example, free-viewing studies of photographic pictures yields evidence for the influence of local visual feature contrasts on fixation directions: Humans prefer to look at locations characterized by local contrasts in terms of color, luminance, or orientation (cf. Itti et al., 1998). This is regarded as evidence for stimulus-driven capture of attention by visual salience, which is basically the summed local feature contrast. Critically, however, in such situations written words notoriously outperform saliency in their attraction of attention (cf. Judd et al., 2009; Borji, 2012). For example, they capture the eyes much more than would be expected based on their relative saliency alone and, thus, require amending the basic salience model (e.g., Borji, 2012). This finding is perfectly in line with a learned and skill-dependent stimulus-driven effect of visual words on attention: Even if currently no linguistic processing is required (as in a free-viewing task), a word that fits to the linguistic skill of reading would trigger an attention shift to the word as part of a stimulus-driven activation of this linguistic skill.

Importantly, the same type of stimulus-driven visual attention in the service of linguistic skills can be observed for other skills than reading. Language production and in particular naming of objects require that the current visual surroundings are taken into account for linguistic processing. For example, if I want to have a sip of milk that is in a jug out of my reach at a coffee table, I could ask a person closer to the milk if she could please hand me the jug. Though I would have some flexibility in how to express my wish, depending on the exact looks of an object, I could not just use any word for the object of my desire. For instance, asking to please hand me the milk container is probably also okay. However, asking for a cup or a spoon would not do the job. These labels would not fit the purpose, my table neighbor would not know that I want the milk jug. In this sense, visual attention to linguistically critical characteristics of my surroundings is necessary for choosing an appropriate label and, thus, visual attention is linked to language production.

Crucially, a tight attentional coupling between verbal labels and objects is not only a logical necessity. It is also the kind of connection observed during language acquisition. In an ingenious study, for example, Smith and Yu (2008), presented pairs of objects to their 12- to 14-months old participants and consistently paired novel verbal labels with only one of the two objects per pair, while the second object was selected randomly from a set of additional novel or yet-to-be-learned labels. In this situation, children preferentially looked at consistently labeled objects, demonstrating that attention – here, the spatial selection of fixated objects – reflected statistical learning and picked up upon the cross-modal visual-verbal probabilities.

## Language Differences and Their Effects on Stimulus-Driven Attention

Instances of language production can vary depending on the language that one speaks (Choi and Bowerman, 1991). While certain features of an object/event are habitually/regularly highlighted in the grammar of one language, they may not be in other languages. To the degree that habitual visual discriminations are required for linguistic productions in Language A but not in Language B, speakers of these languages could differ with respect to their vulnerability to stimulus-driven attention capture by a stimulus in question (cf. Choi et al., 1999). Most obviously, whenever I have to consider my visual environment for an appropriate linguistic expression in Language A, my attention would be directed to the corresponding visual information – that is, the linguistically critical information would be selected as part of my linguistic production skills (cf. Tomasello and Farrar, 1986; Tomasello and Kruger, 1992). This means that attention is habitually shifted to particular features of objects as part of my linguistic practice (cf. Knott, 2012). However, if I, as a speaker of a particular language, now encounter a stimulus habitually fitting to my language skills but outside of the current linguistic/non-linguistic task – that is, when I do not have to produce nor comprehend a fitting linguistic expression and am in an entirely non-linguistic situation – by virtue of the fact that a fitting stimulus could automatically trigger a skill itself, this fitting stimulus could capture attention in a stimulus-driven way and lead to language-dependent visual selection of particular features (cf. Goller et al., 2017).

Goller et al. (2020) recently tested and confirmed exactly this prediction in what may be called an instance of domain-centered research on linguistic relativity (Lucy, 2016). For their tests, they used an established benchmark of stimulus-driven capture of visual attention – the distraction effect by a visual singleton (cf. Theeuwes, 1992). Here, a singleton denotes a visual stimulus that is salient and, thus, stands out by at least one of its features among several more feature-homogeneous non-singletons. For instance, a green apple among red apples would be a singleton among non-singletons. Some singletons can capture attention in a stimulus-driven way, even if entirely task-irrelevant (e.g., Weichselbaum and Ansorge, 2018). For instance, during visual search for a shape-defined target (e.g., for a circle among diamonds) presenting a singleton distractor in a different color than all other stimuli (e.g., a green diamond among red diamonds and one red circle as the target) and presenting this singleton distractor away from the target delays successful search for the target by attracting attention to the singleton distractor (Theeuwes, 1992).

Singleton capture, as we may call this kind of stimulus-driven attention, is not only a consequence of the type of visual information: It can also occur as a consequence of learning (Anderson et al., 2011; Bucker and Theeuwes, 2014; Failing and Theeuwes, 2018). For example, rewarding Color A (say red) on average more than Color B (e.g., green) during a training phase, leads to singleton capture and thus, interference by a color distractor with a previously rewarded color during visual search for a shape target in a subsequent test phase: Even though the color of the target and of just any stimulus in the shape search task is entirely task-irrelevant, that is, does no longer lead to any reward – and participants know all that – presenting the previously reward-associated color as a distractor away from the target delays search, as much as any other singleton would do (Anderson et al., 2011). The learned reward-associated color stands out by its acquired saliency so to say.

Such learning-dependent singleton capture also occurs for a different type of learning, namely linguistic skills. For their tests, Goller et al. (2020) made use of the highly practiced linguistic discrimination of the tightness of the spatial fit between objects in Korean language but not in English or German (Goller et al., 2017; Yun and Choi, 2018). In Korean, speakers ubiquitously use the word *kkita* for a tight fit, for example, when a cap fits on a pen tightly, and they use the words *nehta* or *nohta* for a loose fit, for example, when an olive is loosely surrounded by a bowl (Yun and Choi, 2018). In German, in contrast, such semantic distinctions are possible, but they are not obligatory, nor are they ubiquitously made. Neither is the choice of the suited verb determined by these distinctions, nor is a fit-discrimination an obligatory preposition/particle in German grammar. Thus, only Korean speakers but not speakers of German habitually and obligatorily have to discriminate linguistically between tight and loose spatial fits.

Goller et al. (2020) reasoned that if such skilled linguistic discrimination influences which stimuli or features can capture attention in a stimulus-driven way in non-linguistic tasks, a visual distractor that is presented away from the target and that stands out by the linguistically discriminated feature should be salient and should capture attention in a stimulus-driven way – even when the feature relates to distractors, and not to the target. If so, such attention would interfere with performance even in a non-linguistic color-target search task. Critically, this interference was expected in a group of language users that had to linguistically discriminate the corresponding visual input (here, Korean speakers that are obliged to linguistically discriminate between tight and loose fits for the choice of a correct verb) but not in a group of speakers of a different language without these linguistic obligations (here, German speakers).

**FIGURE 1:**
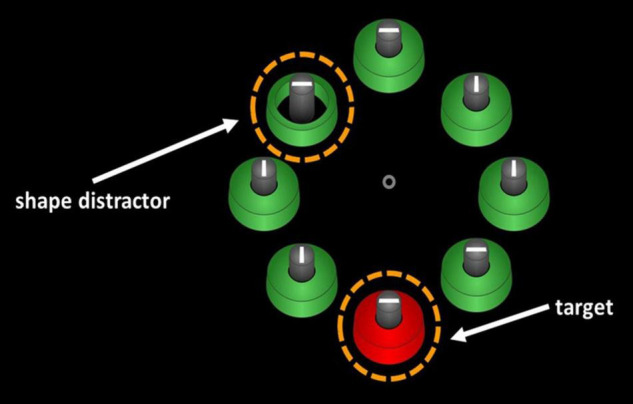
An illustration of the procedure of Goller et al. (2020), Experiment 4. Korean- speaking and German-speaking participants had to search for a color-defined target (e.g., a red target as depicted), and a fit-singleton distractor (e.g., a loose-fit ring among tight-fit rings) was presented away from the target in half of the trials. Compared to a condition without spatial-fit distractor, Korean speakers but not German speakers showed slower search performance for the color targets. This is in line with linguistic relativity, as search for the targets was delayed by stimulus-driven capture of attention toward the spatial-fit singletons only among the Korean speakers that verbally discriminate between tightness of fit levels in an obligatory way, but not among the German speakers that do not have to verbally discriminate different fits.

This expectation was borne out by the results. During search for a color-defined (e.g., red) target among (e.g., green) distractors, presentation of a singleton distractor with a unique spatial fit (e.g., one loosely fitting object among several tight-fitting objects) spatially away from the target delayed visual search among Korean speakers but not among German speakers (Experiment 4 of Goller et al., 2020; see Figure 1, below). In addition, control conditions showed that this stimulus-driven capture of attention by the singleton distractor was not due to a generally larger proneness of Korean speakers to stimulus-driven attention capture. In a control condition, with color-singleton distractors (i.e., a blue singleton-distractor among green non-singleton distractors) that should have been equally salient to both language groups, Korean and German speakers showed the same degree of singleton-distractor interference (Experiment 4 of Goller et al., 2020). In another control condition, Korean speakers showed evidence of stimulus-driven capture by fit-singleton distractors when the distractors were 2D depictions of 3D objects (i.e., pistons within tightly vs. loosely surrounding cylinders) but not when 2D images were used (i.e., disks within tightly or loosely surrounding rings). The latter finding supports the conclusion that stimulus-driven capture by the spatial-fit singletons was language-dependent, as Korean speakers would use tight-fit verbs (e.g., *kkita*) to discriminate the tightness of fit of 3D objects but not of 2D objects. At the same time, these data also demonstrated that Korean speakers were not simply more sensitive to just any type of contextual information (cf. Nisbett and Miyamoto, 2005) – here: of the otherwise task-irrelevant spatial fits–, but just the one that corresponded to highly practiced semantic discrimination in their language. In conclusion, Goller et al. (2020) provide strong evidence of language-specific semantics influencing perception at a stimulus-feature level outside linguistic tasks.

Importantly, in the color-search task, participants did not use a linguistic strategy to solve the task (Baier and Ansorge, 2019): During search for color-defined targets (e.g., a red target among green distractors), participants do not show any signs of verbal rehearsal – that is, visual search performance is not disrupted by the concomitant task of having to rehearse a syllable – and participants’ attention is not captured by visual color words, even if these words denote the searched-for color (e.g., the word red during search for red targets; Baier and Ansorge, 2019).

## How Does Language-Dependent Stimulus-Driven Capture of Attention Account for Linguistic Relativity?

**FIGURE 2:**
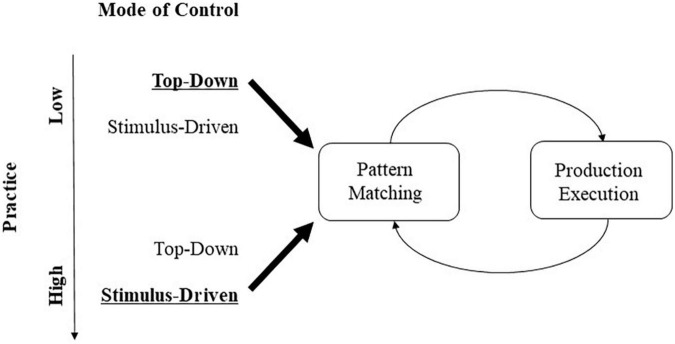
Mode of control – top-down/by goals of the observer versus stimulus-driven – of covert and overt productions in long-term skill memory as a function of skill practice. All productions (e.g., linguistic/attentional, motor) are triggered by pattern matching (see boxes on the left), comparing an input stimulus to a specific template or parameter predefined as a critical precondition for the execution of the production, and the execution of the production itself (see boxes on the right) (Anderson et al., 2004). With practice (downward pointing arrow on the far left), control shifts from top-down, goal-directed selection of the production (top row), to stimulus-driven elicitation of the same production (bottom row). See text for additional explanations.

We have presented and argued for “language-induced automatized stimulus-driven attention” (LASA). According to this argument, attentional selection (of objects, features, or locations) has been repeatedly practiced as part of a linguistic skill to such an extent from early in life that the corresponding forms of selection do no longer require top-down control to trigger such a selection. Instead, with sufficient practice, the corresponding perceptual input can capture attention in a stimulus-driven way – that is, stimuli can provoke their selection for processing by themselves, even outside linguistic tasks. To understand this, let us first look a little closer at skills and their automaticity or proceduralization (Anderson and Lebiere, 1998; Anderson et al., 2004). As Anderson and Lebiere (1998) described in their production model system ACT-R, skills in form of procedural knowledge exist as patterns connecting top-down goals with productions in procedural memory in recurrent feedback loops. Skills consist of sequences of procedures, with to-be-executed productions, where productions cover both overt actions (e.g., grasping an object) and covert processes (e.g., word comprehension) (cf. Anderson et al., 2004). These actions/processes are frequently and habitually practiced and, thus, are part of long-term memory (cf. Ericsson and Kintsch, 1995). As procedures, they follow a general form, consisting of sequences of processing steps that are executed conditionally on the fulfillment of specific eliciting conditions – a process called “pattern matching” in Figure 2 (Anderson and Lebiere, 1998). For example, in language production, a speaker would first look at the critical characteristics of an event to produce a sentence describing what she sees (cf. Slobin, 1996). Imagine a speaker of English who registers the durative nature of an illustrated event in a book and marks the progressive aspect on a fitting verb, for instance, “the dog was running from the bees” (cf. Slobin, 1996). Importantly, and in contrast to what Slobin (1996) believes, pattern matching and, hence, attention shifts to the corresponding characteristics of an image would not only run off in a top-down controlled fashion only, for example, when an intention to communicate requires this, but also, following repeated language practice, a stimulus as a fitting input could trigger the production. That is, the durative nature of an event would attract attention – without intervening top-down control, therefore, practice facilitates stimulus-driven shifts or capture of attention. As skills are frequently or habitually practiced, they become automatized (or proceduralized): When a skill is originally acquired, it typically requires exerting top-down control to get started. However, practicing a skill means that control about what to do next in a sequence of overt motor responses or covert processing steps is delegated more and more to the stimuli that are used in the course of a skill’s pattern matching process (Neisser and Becklen, 1975; Anderson and Lebiere, 1998; Anderson et al., 2004). Thus, with practice, stimuli used in pattern matching take over the role of initiating a procedure.

In the case of the sensation of a particular sensory pattern triggering an attention shift that originally served a correct linguistic production, this is really not such a wonder, as above we have already emphasized that such visual-verbal pattern-label associations form an important basis of language acquisition in the first place (Smith and Yu, 2008).

At this point, a related clarification is in order that might be responsible for reservations against our suggested hypotheses. We have emphasized that language comprehension and production are skills. Therefore, they are part of the (more implicit) procedural memory of humans (Ullman, 2001; Lum et al., 2012). This means that (1) the selectivity underlying perception implied by a particular language is not entirely under voluntary top-down control and (2) these forms of selective processing often go unnoticed by humans from an introspective, first-person perspective. In addition, this also means that language effects on the stimulus-driven forms of perceptual input selection are possible and even likely, as they are typical of well-practiced skills and procedures in general (Allport, 1987) and of language in particular (Pulvermüller and Shtyrov, 2006; Kotz and Schwartze, 2010).

However, there might be more to language as a skill than the types of bidirectional associations between verbal labels and attention shifts that we described. Most critically, language is characterized by higher-order regularities besides the discussed item-adjacent visual-verbal dependencies. There are many more language-specific regularities concerning non-adjacent item dependencies that are reflective of a recursive or hierarchical language-inherent structure, and, importantly, these higher-order regularities could be more typical of natural languages than the simple visual-verbal dependencies which are at the focus of our argument (Fitch and Friederici, 2012; Jager and Rogers, 2012; Fitch and Martins, 2014). We do not claim that all of these language-inherent statistical characteristics need to be equally easily triggered by the mere presence of a matching pattern. It might well be that stimulus-driven language-specific skill elicitation is restricted to the somewhat simpler associations between sensory inputs and originally linguistic attention shifts that we described above.

This brings us to the interesting question about the “complexity” of these associations and the type of representational system that might be used to represent these types of skills. First of all, we want to emphasize that a degree of processing demand is implied by the cross-modal nature of the visual-verbal links that we described. A representational system sensitive to the statistical or temporal regularities present within a single modality would, thus, be insufficient to represent the corresponding information (cf. Keele et al., 2003). This makes it more likely that the corresponding knowledge is represented in what Keele et al. (2003) called the “ventral system,” possibly, with the Inferior Parietal Lobe as the area of multi-modal convergence in which the types of spatial tight- versus loose-fit relations that Goller et al. (2020) investigated could be represented (cf. Kemmerer, 2006). This system would be sensitive to the task relevance of the associated material – here the visual-verbal pairs connecting fitting sensory input with a corresponding verbal label – and, hence, its proper functioning could even sometimes be vulnerable to the characteristics of a non-linguistic test task. For example, it might simply be difficult to observe the corresponding language-dependent attention shifts in non-linguistic tasks if these test tasks would require or even only offer the acquisition of even simpler (e.g., within-modality) item associations. Here, we can see that there are reasons for the possible failure of tests of linguistic relativity beyond the ones discussed by Slobin (1996), who speculated that such non-linguistic tests could fail because of the language-specific nature of linguistic discriminations that has little connection to other sensory and motor discriminations. On the contrary, we would argue that as the building blocks for linguistic skills are relatively similar to that of sensory and motor skills, a point that is particularly true of attention shifts to objects, their features or characteristics, there is plenty of opportunity for the alternative usage of the same non-linguistic part devices in non-linguistic tasks that could potentially block or allow their usage as according to a linguistic skill in these very same tasks.

Here, we want to conclude that the thereby assumed more gradual nature of the involved skills and representations as being neither perfectly sensory/motor, nor fully linguistic, is also better in line with the type of proto-conceptual representations that are obviously driving perception from infancy onward (Xu, 2019). In particular, Xu (2019) came to the conclusion that perceptual discriminations of infants already show characteristics of linguistic representations, such as (partial) categorization and (imperfect) enrichment of situations with organismic beliefs, that are not yet at an adult level but that would certainly also make it difficult to keep up the impermeable boundary between the linguistic and the sensory sphere that seems to have dominated the thinking of the great theoreticians for so long (cf. Piaget, 1954; Chomsky, 1987; or Slobin, 1996; Pylyshyn, 1999).

### Relationship Between Attention and Perception

Of interest for the theory of linguistic relativity is now the relation between attention and perception. The argument is basically that perception depends on attention and, thus, linguistic influences on attention can literally shape our view of the world. Inherent to this argument for linguistic relativity through language-dependent stimulus-driven attention is the conviction that attention precedes perception and, thus, shapes what humans perceive. Since long, it has been assumed that without attentional selection, perception is not possible (Titchener, 1908; Di Lollo et al., 2000). Although this position might be too extreme and there might be situations in which perception is possible without attention, attention as the selection of particular stimuli, features or locations is at least abundant. Attention can at least facilitate and, thus, modulate perception, when manipulated by the experimenter (Scharlau, 2002, 2004; Scharlau and Neumann, 2003). For example, shifting attention to the position of a visual stimulus, in advance of this stimulus, can speed up this stimulus’ perception as reflected in its subjectively apparent temporal precedence relative to a concomitant second stimulus that does not benefit from a like shift of attention (Scharlau, 2002). In other words, of two simultaneous stimuli, the one that we attend to first is perceived earlier: It seems to precede the stimulus to which we do not attend. Thus, perception – here of the time at which a stimulus is perceived – is shaped by attention. Importantly, in line with a role of attention for perception, this selection of perceptual input can occur independently of and, thus, prior to the perceiver’s awareness of a stimulus (Scharlau, 2002; Scharlau and Ansorge, 2003). This temporal sequence of attentional selection prior to perceptual awareness of a stimulus is in line with the modulating role of attention on perception. In addition, attentional selection of one stimulus, feature, or event can come at the expense of missing out on alternative stimuli, features, or events (Mack and Rock, 1998; Simons and Chabris, 1999; Horstmann and Ansorge, 2016). In this way, language-induced stimulus-driven attention (LASA) is also in a position to determine what exactly a human observer can perceive.

### How Much Does Language Determine Humans’ Perception of the World?

As we have argued, as a skill, language is closely linked to attention in a way that the domains of language and perception are not such distinct unconnected spheres. However, we are not certain how much this extends to differences for human perception as a whole. The reasons for this are twofold. First, different languages share commonalities. For example, Yun and Choi (2018) proposed that languages share a common set of spatial features (e.g., containment, support, degree of fit) but that they differ in the degree to which they highlight those features in their semantic system. The varying degrees of commonalities also create a huge overlap in the way humans perceive objects, regardless of the particular language they speak. Second, the language-specific effects on stimulus-driven attention and perception are in general similar to other forms of practice-dependent long-term memory effects. Each skill that humans acquire also entails some forms of skill-dependent sensitivities and insensitivities for the selection of particular skill-implied perceptual inputs (cf. Johnson, 2013). Language is, thus, not the only way in which practice leads to a change of attention and perception. As a consequence, many other human skills, such as walking, grasping, driving, or eating, can all have an impact on how we humans attend to objects and, thus, perceive them. These non-linguistic skills provide a rich source for both language-independent commonalities and differences in the way humans allocate attention and perceive the world.

Nevertheless, in the present review, we highlighted that language alters the way humans’ attention is attracted by different stimuli and features. As each language has its own semantic system that systematically highlights a specific set of features or feature differences, which may differ – and often do – from other languages, attention to those language-specific features taken together can contribute to significant differences in the way speakers of different languages look at the world (cf. Thierry et al., 2009; Lupyan, 2012).

## Conclusion

Critics of linguistic relativity used task-dependent top-down attention to verbal concepts to explain away language-induced effects and, thus, would not accept that language can influence attention/perception in language-independent non-linguistic contexts. In this paper, we make a counter argument with a set of well-established processing mechanisms showing intimate interaction between language and perception/attention, and demonstrate that linguistic relativity is possible and even likely. In our proposed “language-induced automatized stimulus-driven attention” (LASA) explanation, attention – indeed – plays a very important role during language acquisition and processing, and in fact language and attentional skills are highly interconnected, which are then practiced jointly throughout life. These extensively rehearsed coevolved skills then subsequently lead to stimulus-driven, automatic attention capture by fitting stimuli. In this way, linguistic influences generalize, for example, to the visual selection and perception of specific objects or features, even in language-independent tasks.

## Data Availability Statement

The original contributions presented in the study are included in the article/supplementary material, further inquiries can be directed to the corresponding author/s.

## Ethics Statement

Ethical review and approval was not required for the study on human participants in accordance with the Local Legislation and Institutional Requirements. The patients/participants provided their written informed consent to participate in this study.

## Author Contributions

UA drafted the manuscript. DB and SC revised it. All authors contributed to the article and approved the submitted version.

## Conflict of Interest

The authors declare that the research was conducted in the absence of any commercial or financial relationships that could be construed as a potential conflict of interest.

## Publisher’s Note

All claims expressed in this article are solely those of the authors and do not necessarily represent those of their affiliated organizations, or those of the publisher, the editors and the reviewers. Any product that may be evaluated in this article, or claim that may be made by its manufacturer, is not guaranteed or endorsed by the publisher.
